# Longitudinal Analysis of Vaginal Microbiome Dynamics in Women with Recurrent Bacterial Vaginosis: Recognition of the Conversion Process

**DOI:** 10.1371/journal.pone.0082599

**Published:** 2013-12-20

**Authors:** Janet A. Lambert, Susan John, Jack D. Sobel, Robert A. Akins

**Affiliations:** 1 Department of Biochemistry, Wayne State University School of Medicine, Detroit, Michigan, United States of America; 2 Division of Infectious Diseases, Wayne State University School of Medicine, Detroit, Michigan, United States of America; University of Iowa Carver College of Medicine, United States of America

## Abstract

Bacterial vaginosis (BV) affects ∼30% of women of reproductive age, has a high rate of recurrence, and is associated with miscarriage, preterm birth, and increased risk of acquiring other sexually transmitted infections, including HIV-1. Little is known of the daily changes in the vaginal bacterial composition as it progresses from treatment to recurrence, or whether any of these might be useful in its prediction or an understanding of its causes. We used phylogenetic branch-inclusive quantitative PCR (PB-qPCR) and *Lactobacillus* blocked/unblocked qPCR (Lb-qPCR) to characterize longitudinal changes in the vaginal microbiota in sequential vaginal self-swabs from five women with recurrent BV, from diagnosis through remission to recurrence. Both patients with acute BV samples dominated by *G. vaginalis* recurred during the study with similar profiles, whereas the three patients with acute BV samples dominated by other anaerobes did not recur or recurred to an intermediate Nugent score. *L. iners* dominated remission phases, with intermittent days of abnormal microbial profiles typically associated with menses. The exception was a newly discovered phenomenon, a sustained period of abnormal profiles, termed conversion, which preceded symptomatic acute BV. Species known to have antagonistic activity towards *Lactobacillus* were detected in pre-conversion samples, possibly contributing to the decline in *Lactobacillus*. Lb-qPCR scores define two categories of response in the initial post-treatment visit samples; scores <5 may correspond with poor response to treatment or rapid recurrence, whereas scores >8 may predict delayed or no recurrence. Amsel criteria or Nugent scores did not have this potential predictive capability. Larger studies are warranted to evaluate the prognostic potential of detecting conversion and poor Lb-qPCR scores at the post-treatment visit of recurrent BV patients.

## Introduction

Bacterial vaginosis (BV) is a common vaginal infection associated with major complications including adverse reproductive health outcomes [1], [2] and increased risk of of HIV-1 acquisition [3]–[8] and other sexually transmitted infections [9]–[11]. The vaginal microbial community, or microbiota, in women with BV is significantly altered from the normal healthy status of dominance by *Lactobacillus* sp. Specifically, *L. crispatus* and *L. jensenii* [12], [13] are consistently either at low titer or absent in women with BV but *L. iners* is only partially reduced in titer [14]. In BV, lactobacilli are displaced by a variety of anaerobic species, inconsistently including *Gardnerella vaginalis*, *Prevotella* spp., *Mobiluncus* spp., *Atopobium vaginae,* as well as other anaerobic organisms, including BV-associated bacterium (BVAB) −1, −2, and −3 [15]–[18]. The etiology of BV remains unknown, hence cure rates remain unsatisfactory and recurrence rates are extremely high [19]–[21]. Despite the availability of new molecular tools to amplify and recognize previously uncultivated bacterial species and the explosion of studies revealing some of the enormous microbial diversity of the vaginal microbiome in healthy, asymptomatic, and acute BV patients [22]–[43], clarification of the sequence of events in the development of BV has not been forthcoming.

Recurrence is a key problem in BV. For example, one study found 58% of 121 women with BV, who were successfully treated with metronidazole, recurred within one year; 69% returned to abnormal vaginal profiles [19]. Arguments that recurrence occurs by relapse include increasing microbial resistance rates [44], although this may only apply to clindamycin [45], [46], and that recurrence rates are higher in asymptomatic women with higher Nugent scores, presumably reflecting more complex flora [47]. Arguments for recurrence by reinfection include that recurrence rates are 50% lower in women who abstain from coitus after treatment or consistently use condoms [48] and are significantly higher in women after unprotected coitus. It is not clear if vaginal species in varying proportions represent subgroups that impose varying risks of complications or of symptoms, and if they play a role in conversion of the healthy vaginal microbiome. A small study showed strong predictive value of prevalent Gram-positive cocci in pretreatment Gram stains of BV patients for rapid recurrence [49].

There are many published longitudinal studies, but most sampled at long intervals, often weeks or months [14], [50]–[64]. Although much valuable information can be gleaned from these studies, they cannot show rapid fluctuations that were demonstrated in studies that used daily vaginal swabs over at least a portion of the study interval [65]–[70]. An under-appreciated consequence of single-sample studies, which are the norm, is that they capture glimpses of dynamic processes at unknown stages. However, none of these studies sampled with sufficient frequency or depth enough to capture sequential changes in the vaginal microbiota as BV recurs.

In the present study, we report and compare the detailed longitudinal microbial profiles of five women with histories of recurrent BV, using qPCR methods we recently described [71]. Two of the five patients recurred with acute BV, slowly in one case, more rapidly in the other case, following metronidazole therapy. Two did not recur during the study, and one showed a poor response to therapy and presented with an intermediate Nugent score. Data show high levels of variation of target species during recurrence, and differences in profiles between acute BV samples of the five patients, which change in sequential episodes. More importantly, data suggest that incomplete restoration of *Lactobacillus* sp. after therapy predicts poor outcome, and that the microbiome can undergo a newly described event termed conversion, the decline in *Lactobacillus* and rise of replacement species, days to weeks before symptomatic BV.

## Materials and Methods

### Patients

Five African American participants were followed at the Vaginitis Clinic at Wayne State University and had been treated for recurrent episodes symptomatic bouts of BV. Patients were enrolled after protocol explanation and written informed consent was obtained. The study was endorsed by the Human Investigational Review Board of Wayne State University. At the time of enrollment, patients were diagnosed with florid symptomatic acute BV, characterized by the presence of at least 3 of the 4 Amsel criteria (homogeneous vaginal discharge, pH elevated above 4.5, clue cells>20%, positive whiff test) [72] and Nugent scores of at least 8 [73] (Table 1, details in Table S1). Patients were treated with a metronidazole, clindamycin, or tinidazole regimen and returned within three weeks with a clinical cure by Amsel criteria (zero criteria positive) and Nugent scores of 0, except Patient 5, who had an intermediate Nugent score of 4. At this immediate post-treatment visit, patients were given Catch-All Sample Collection Swabs (Epicentre Biotechnologies, Madison, WI) and 15 mL conical tubes for vaginal specimen self-collection, instructed as in Methods S1. Patients were seen at the Vaginitis Clinic and evaluated monthly at which time vaginal swabs were collected. More information is detailed in Methods S1. Samples from a previously characterized group of recurrent BV patients, sampled before and after treatment [74], were also evaluated by Lb-qPCR.

**Table 1:** Profiles and treatment regimens for recurrent BV patients.

		acute BV				post-treatment			recurrence		
	Age	day	A	N	treatment	day	A	N	day	A	N
**P1**	40	−277	4	10	n/a	−259	0	0	0	4	9
**P1**	40	0	4	9	750 mg metronidazole suppositories/day	10	0	0	94	3*	8
**P2**	26	0	3*	9	oral metronidazole 500 mg bid	7	0	0	35	4	8
**P2**	26	35	4	8	2*500 mg metronidazole suppositories/day	41**	n/a	n/a	152**	n/a	n/a
**P3**	35	0	4	10	2% clindamycin	21	0	0	None as of day 175		
**P4**	24	0	4	9	tinidazole 500 mg bid	7	0	0	None as of day 35		
**P5**	32	0	4	8	500 mg metronidazole vaginal suppositories	21	0	4	77	4	4

Note: Day 0 refers to day of enrollment as a longitudinal patient. Treatments were all for 7 days. A =  Amsel criteria, number positive; N =  Nugent score. n/a =  not available. *  =  Clue cells present but not above 20%; **  =  not clinically confirmed.

**Figure 1:**
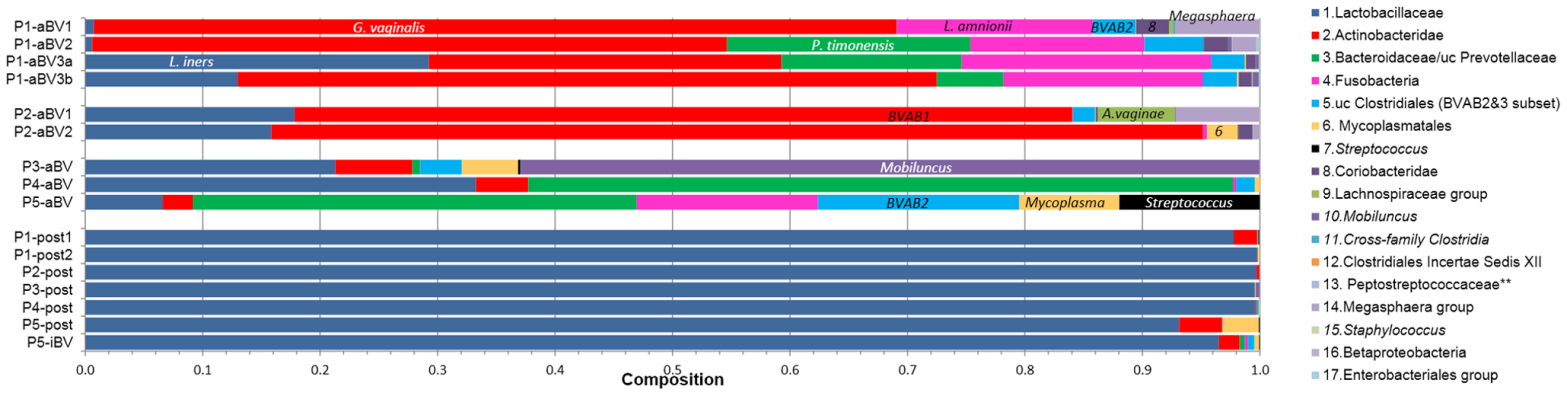
PB-qPCR generated microbial profiles of acute and post-treatment vaginal swabs of patients with histories of recurrent BV. Data is also provided as Table; these and the conversions to relative titers as described [71]. Patient 1 (P1) was sampled at 3 separate acute BV (aBV) episodes; 3a and 3b are samples of the 3^rd^ episode taken 5 days apart. P1 and P2 recurred during the study; P3 and P4 did not. P5 responded poorly and was ultimately diagnosed with BV at an intermediate Nugent score, 4 (P5-iBV). uc  =  uncultured.

### Sample processing, DNA extraction, and qPCR

Microbial gDNA was purified from swabs in 1–2 ml saline or lysis solution by a high SDS/alkaline lysis – phenol extraction protocol as described previously [71] and resuspended in 200 µL TE (10 mM Tris, pH 8, 1 mM EDTA). The lysed bacterial gDNA is stable for over one month at room temperature in lysis solution buffer (data not shown) and this buffer prevents changes in titer due to possible stability issues with different strains of bacteria in saline. An aliquot of purified DNA was assayed by qPCR with 18 universal and phylogenetic branch-inclusive (PB) primers and PCR conditions as described previously [71]. Additional primers, targeting the *Enterococcus* genus and its cytolysin gene CylL_L_, are characterized in Table S2. Each PB-primer targets a branch of the phylogenetic tree, from whole phyla to family or genus, and is far more inclusive than species-specific primers. Relative *Lactobacillus* composition of the vaginal microbiome was determined by dividing the Lactobacillaceae titer by the sum of all PB-primers and by using our LB-blocker approach [71]. Briefly, our LB-blocker approach uses the difference between the quantitative cycles (Cq) of two parallel qPCR reactions with universal primers targeting the 16S rRNA gene, when one of the reactions is in the presence of *Lactobacillus* spp.-specific blocking oligomers (LB-blockers) that have been chemically modified to prevent extension and that partially overlap the universal primer binding site, effectively rendering the *Lactobacillus* DNA “invisible” to the universal PCR, even when *Lactobacillus* gDNA is present at great excess compared to non-*Lactobacillus* gDNA, as is common in healthy and post-treatment samples. PB-primers amplicons were selectively sequenced to confirm they were correctly targeted and to identify the dominant species. These were “cleaned” enzymatically as described [71], and Sanger sequenced at Functional Biosciences (Madison, WI). Sequences were uploaded to Ribosomal Database Project II using its Pipeline function; species were identified based on phylogenetic trees constructed in Molecular Evolutionary Genetics Analysis (MEGA5) software (2, 3).

### Calculations

Cq values from qPCR reactions were converted to molecules per reaction by comparing sample Cq values to a standard curve from the same run of Cq values derived from dilutions of an amplicon of known target of known concentration. Molecules per reaction were converted to cells per swab, assuming an average of 5 ribosomal genes per cell and proportioned to the ratio of the volume of the sample used in the DNA prep and the amount of sample used in the qPCR reaction. Delta Cq (ΔCq) values of LB-blocked reaction pairs [71] were calculated as Cq (blocked sample) – Cq (unblocked sample). Differences between patient groups were compared using the t test in (GraphPad software).

**Figure 2:**
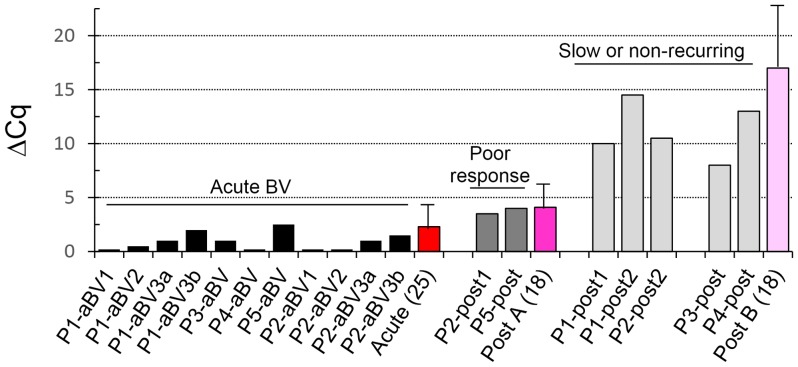
ΔCq values in samples from RBV patients at visit 1 (acute BV, black bars) versus post-treatment visit 2 (grey bars). Patients who recurred rapidly recurring or responded poorly responding (P2, P5) are shown in dark grey; patients who did not recur or did so slowly are in light grey (P1, P2 after her 2^nd^ recurrence, P3, P4). Red to pink bars represent averages of a separate collection of patients (numbers in parentheses) who were only sampled at the initial acute BV and/or the post-treatment visits, with indicated standard deviations.

## Results

### Overview of vaginal profiles of 5 patients (P1 to P5) with acute BV and after treatment

Figure 1 illustrates the profiles of 5 patients with histories of recurrent BV at enrollment with BV and after treatment. We analyzed samples from between one and three acute BV (aBV) episodes from each patient, as well as post treatment 7 to 21 days after the first diagnosis. Results are phrased here as PB-primer target (species by sequence). All post-treatment samples were restored to dominance by Lactobacillaceae; predominantly *L. iners* or transiently *L. jensenii*; some samples were co-dominant for these as indicated by sequence polymorphisms. Acute BV samples fell into two groups: P1 and P2 (recurring patients) were dominantly Actinobacteridae (*G. vaginalis*); in contrast non-recurring patients P3 and P4, nor for P5, who responded poorly to therapy, were dominated by phyla other than Actinobacteridae. Sequential BV profiles from the same patient had differences, but were more similar to each other than to BV profiles of the other patients. BV samples were positive for 76–100% of the PB primers (ranging from 6–17 tests among individual samples) at subdominant or low levels, more evident in Table S3. Post-treatment samples were still positive for 67–94% of the PB-primer targets, typically at much lower levels than the BV samples.

Patient 1 (**P1**) is characterized as a slowly recurring BV patient, who had 3 acute BV (aBV) episodes over 371 days (Fig. 1). At her first episode (P1-aBV1), she presented with acute BV, dominated by Actinobacteridae (*G. vaginalis*), but also having diverse species including sub-dominant (2–14%) Fusobacteria *(Sneathia sanguinegens), Megasphaera/Dialister/Veillonella* (*Megasphaera genomosp*. type 1), uc Clostridiales-*BVAB2/3* (*BVAB2*), and Coriobacteridae (*Atopobium vaginae)*, and 11 other groups at lower titers. Her profile was very similar at her second BV episode, most notably different only in her increased proportion of Bacteroidaceae/Prevotellaceae (*Prevotella timonensis*). Her third BV episode (divided here into the clinical confirmed episode, aBV3b, and her self-swab 5 days earlier, aBV3a), differed in having higher proportions of Lactobacillaceae (primarily *L. iners*) and Bacteroidaceae/Prevotellaceae (*P. timonensis*), and reduced levels of *Megasphaera* (*Megasphaera genomosp*. type 1). Comparing the P1-aBV3a to P1-aBV3b, *G. vaginalis* increased further at the expense of *L. iners*, and Lachnospiraceae (*BVAB1*) increased ∼500 fold (Table S3).

After both first and second BV episodes (P1-post1 and -post2), she responded well to high dose vaginal metronidazole treatments, becoming Amsel negative and having Nugent scores of 0. Consistently, both samples were >97% Lactobacillaceae (primarily *L. iners*). Actinobacteridae (*G. vaginalis*) was incompletely eradicated in P1-post1 at 2%, but was reduced by another ∼100-fold in P1-post2. The level of reduction of *G. vaginalis* in this slowly recurring patient is similar to levels seen in non-recurring patients P3 and P4.

**Figure 3:**
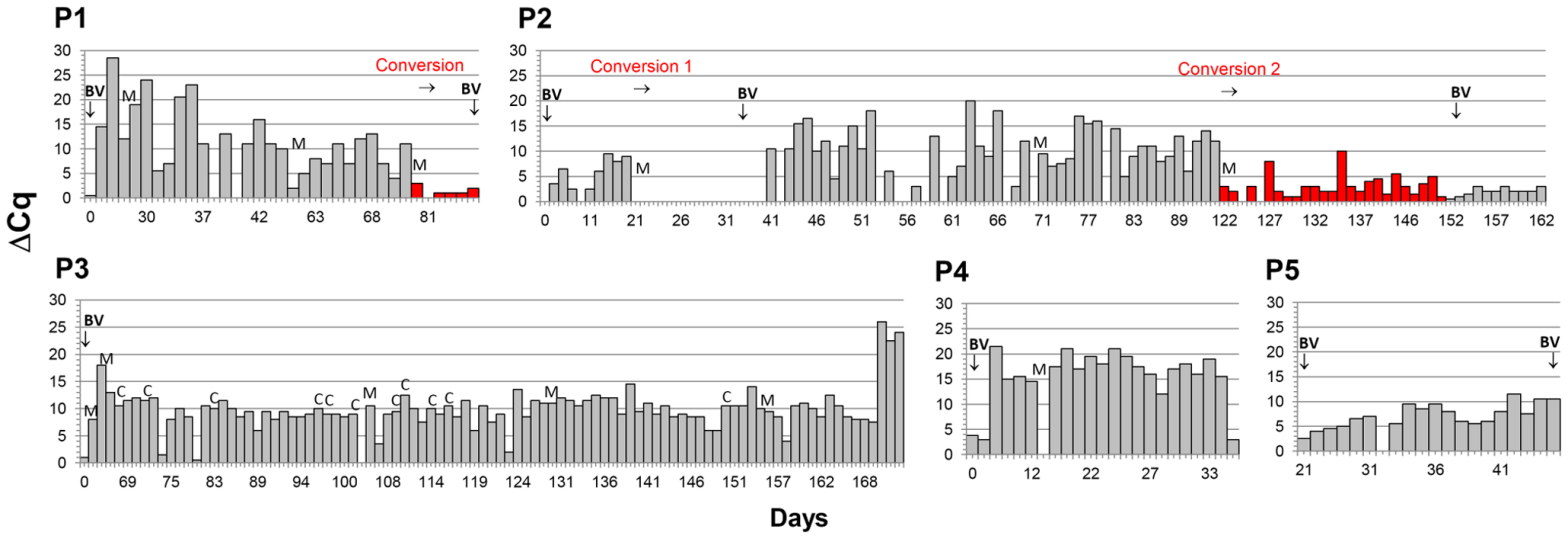
ΔCq values define conversion events before acute BV. Conversions (red bars) ΔCq values persistently <5 occurred 10–40 days before symptomatic BV in recurring patients P1 and P2, but not in non-recurring patients P3 and P4. Values remain <5 for most of the interval in P5. BV =  acute BV by Nugent and Amsel; M =  menses; C =  coitus.

Patient 2 (**P2**) is characterized as a rapidly recurring BV patient. The first post-treatment responses of P2 after successful oral metronidazole treatment resulted in an Amsel negative status and Nugent score of 0 (Fig 1, P2-post1). Lactobacillaceae (*L. iners*) rose to similar levels of dominance compared to P1, Actinobacteridae (*G. vaginalis*) fell to <0.5%, and 6–8 other targets were seen at under 0.1%. After recurrence at day 35, similar response resulted from her second treatment, 2×500 mg metronidazole suppositories daily for 7 days, but in this case her recurrence occurred more slowly than the first, approximately 117 days post-treatment based on self-reporting, since she did not revisit the clinic.

Patient 3 (**P3**), in contrast to P1 and P2, did not recur during the 172 days after enrollment. She was successfully treated with clindamycin to progress from Amsel positive and Nugent 10 to Amsel negative and Nugent 0. Her acute BV sample (Fig. 1, P3-aBV), was notably different from P1 and P2 in that her Actinobacteridae (*G. vaginalis*) component was ∼10 fold lower, and her *Mobiluncus* component (*M. mulieris*) was >20 fold higher. After treatment (Fig. 1, P3-post), Lactobacillaceae (*L. iners*) rose to extreme dominance as expected, and Actinobacteridae (*G. vaginalis*) decreased 10–100 fold lower than P1 or P2, and except for *Mobiluncus* at 0.4%, all other targets were below 0.01%.

Patient 4 (**P4**) was similar to P3 in that she did not recur, but was only followed for a month before being dropped from the study due to pregnancy. However, at enrollment with acute BV, her initial sample P4-aBV1 was atypical, characterized by low Lactobacillaceae (*L. iners*) and low Actinobacteridae (*G. vaginalis*), as seen in non-recurring P3, but high levels of and Mycoplasmatales (mixed *Mycoplasma* spp.). She responded well to tinidazole treatment, with high dominance by *L. iners*, and the 7 non-*Lactobacillus* targets that were detected were under 0.1% (Table S3).

Patient 5 **(P5)** recurred in two and a half months, but only to an intermediate level, Nugent 4. At enrollment with acute BV, P5-aBV had a unique profile among the group, characterized by low Lactobacillaceae (*L. iners*), low Actinobacteridae (*G. vaginalis*), but high levels of Bacteroidaceae/Prevotellaceae (*P. timonensis*), Clostridiales-*BVAB2/3* (*BVAB2*), Fusobacteria (*S. sanguinegens* variant), Mycoplasmatales (*M. hominis*), and *Streptococcus* (*S. agalactiae*). After treatment, P5 had a higher level of Actinobacteridae (*G. vaginalis*) than the other patients and a Nugent score of 4, and therefore is characterized as having only a partially successful response. She reported odor or discharge throughout the month, until she was diagnosed as with BV by Amsel, at an intermediate Nugent score, 4, by day 77.

**Figure 4:**
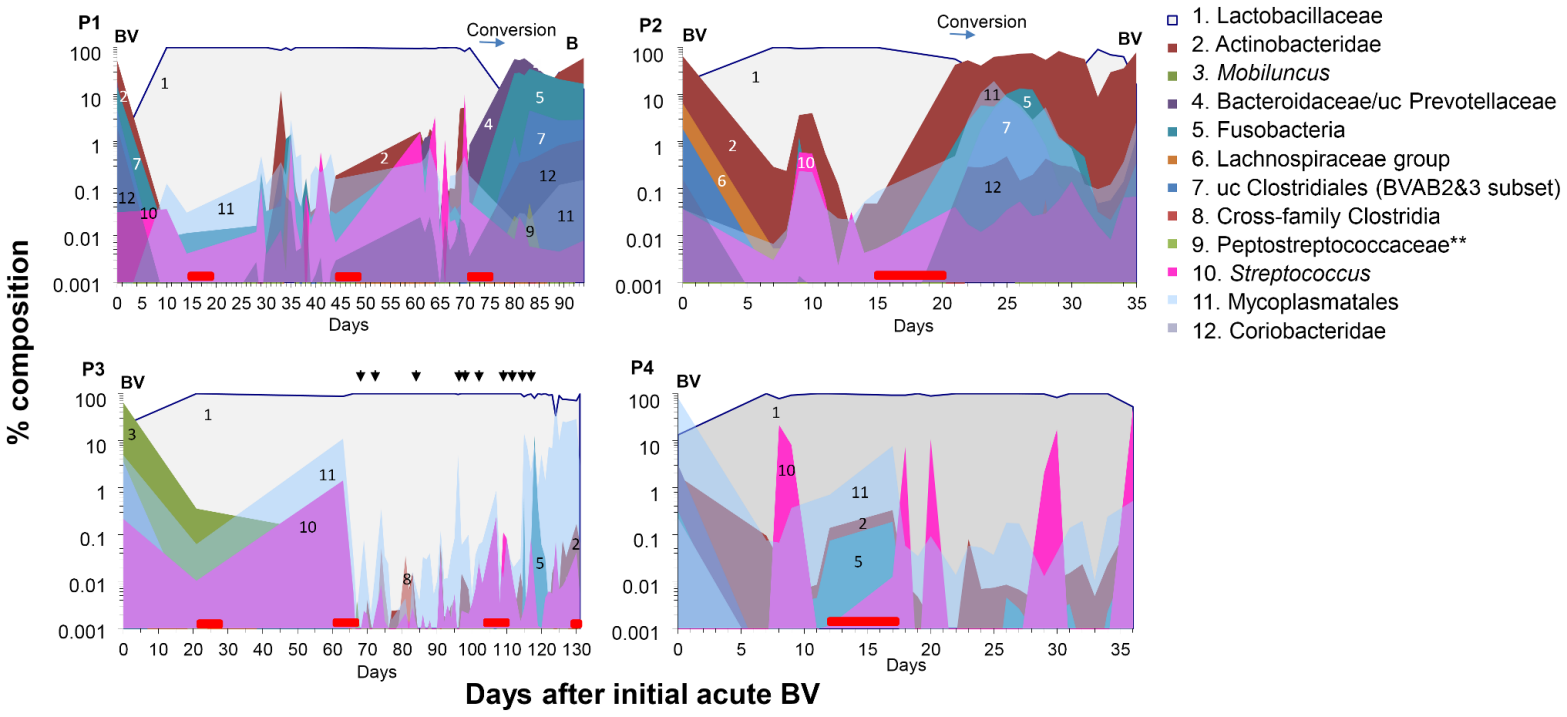
Microbial profiles of near-daily vaginal swabs from patients with histories of recurrent BV, characterized with 11 PB-qPCR targets (legend). Data are converted to % total titers and depicted on a log scale. Top panels of P1 and P2 show the expected rise to dominance of *Lactobacillus* after treatment, and conversions before acute BV. In both patients, sharp increases are seen in *G. vaginalis*, (2), *Prevotella* (4), *L. amnionii* (5), *BVAB2* (7), and *Mycoplasma* sp. (11). Patients P3 and P4, who did not recur, show sustained dominance of *Lactobacillus* after treatment, and their non-*Lactobacillus* populations remain generally at <1%, with frequent transient spikes. Red bar  =  menses;↓ =  coitus. Data is also presented as Table S4.

### Relative *Lactobacillus* content as an overview of vaginal microbiome status

We previously demonstrated that oligomers that specifically block amplification of *Lactobacillus* (LB-blockers) in an otherwise universal PCR of the 16S rRNA gene allow detection of the subdominant species in healthy or post-treatment BV patients (Lb-qPCR) [71], such as those barely visible in Fig. 1. It follows that the Cq of BV samples in Lb-qPCR, in which *Lactobacillus* is sub-dominant, should change slightly or not at all in blocked versus unblocked reactions, and the Cq of healthy samples, often composed of >99% *Lactobacillus*, should shift up by more than 6–7 cycles (2^6^ = 64; 2^7^ = 128) with LB-blocking. Consistently, the average delta Cq (ΔCq) value between blocked and unblocked samples taken from our 5 patients during acute BV, was 1, whereas the average ΔCq was 11 in the post-treatment samples. Furthermore, ΔCq values were intermediate (∼4) in post-treatment samples in patients who recurred rapidly or had an incomplete response (P2, P5), and were much higher, 8–15, in the remaining patients who either recurred more slowly or not at all (Fig. 2).

Lb-qPCR analysis was also performed on a separate, larger patient group of recurrent BV patients before and after metronidazole treatment (Fig. 2). Data confirm that the ΔCq scores were consistently low (2.3±1.6) in acute BV samples (Nugent average 8.8±1.0), and fall into two distinct categories after treatment: Post A, ΔCq scores averaging 4.1±2.2 (Nugent average 2.2±4.0) versus Post B, ΔCq scores averaging 17±6 (Nugent average 0.8±1.2). An out-group of 28 patients with no history of BV had an average ΔCq value of 16.0±5.0 (data not shown). Differences were not significant between the Post B and healthy groups (p = 0.5437), small but significant comparing acute BV and the Post A groups (p<0.0034) and significant and large comparing Post A and Post B (p<0.0001; 95% C.I. 9.8–16).

### Conversion of vaginal microbial profiles preceding acute BV

ΔCq was used to track the overall compositions of daily vaginal samples of our five patients as they either recurred with BV or maintained a nonrecurring status (Fig. 3). Values fluctuated on a daily basis, but several useful trends were noted. P3 and P4, who did not recur, maintained high ΔCq values (i.e., low titers of non-*Lactobacillus* sp.) throughout their post-treatment histories, averaging 10 and 15, respectively. In these non-recurring patients, lower ΔCq values were seen only sporadically for a single day, and often associated with menses or 2–3 days after coitus. In contrast, rapidly recurring P2 showed an initially weak response to treatment, average ΔCq  = 5, which dropped to near 0 after menses beginning at day 21, and remained near 0 for 2 weeks, at which time BV was clinically diagnosed. P2 responded somewhat better to her second treatment from day 41; her ΔCq sustained an average of 9, until after menses at day 121. This interval, however, was unstable; there were several days with sequential low ΔCq values <5, and half of the samples had ΔCq values <10. After her final menses, ΔCq values averaged <3 and never rose above 10; eventually self-reported symptoms of BV recurred. We define these sustained intervals of low ΔCq values, days 21–35 and 121 onward, as conversion, reflecting large declines in *Lactobacillus* content and take-over by a variety of non-*Lactobacillus* species.

P1 recurred but more slowly than P2 in her initial recurrence (3 months versus 1 month). Consistently, P1 had ΔCq values of ∼20 for the first month, versus 5 for P2, and P1 remained symptom-free for ∼90 days, versus 35 days for P2. However, ΔCq values for P1 trended downward after successive menses (averages 22, 12, 8) until conversion after her last menses, at which time her ΔCq averaged 1.

All conversions in P1 and P2 immediately followed menses and all preceded BV by more than a week. No conversions were seen in non-recurring patients P3 and P4. Not all menses are associated with conversion, but in P1, menses associated with progressive declines in average ΔCq values.

### Conversion events

**P1.** Conversion events were characterized in more detail by PB-qPCR (Fig. 4). In P1 at day 80, after menses, there was a 20-fold drop in *Lactobacillus*, concomitant with a 100-fold increase in Actinobacteridae (*G. vaginalis*). Simultaneously, Bacteroidaceae/Prevotellaceae (*P. timonensis*), Coriobacteriadae (*Atopobium vaginae*), Fusobacteria (*Leptotrichia amnionii*), and uc Clostridiales-*BVAB2/3* subgroup (BVAB2) increased 10–1000 fold. Smaller changes in *G. vaginalis* and BVAB2 began before conversion, around day 60, preceded by and associated with spikes and species shifts in *Streptococcus*, from the *S. anginosus* group (*S. anginosus, S. constellatus,* and *S. intermedius*) to the *S. mitis* group (*S. mitis, S. cristatus, S. infantis, S. oralis,* and *S. pneumonia*), groups as defined [75], [76], not identifiable further by the amplicon we sequenced. Notably, the rise in *BVAB1* occurred at the end of conversion, at day 94.

The ΔCq metric alone reliably defined conversion events, and all 3 were confirmed and further characterized by PB-qPCR data. The initial BV population, dominated by Actinobacteridae (*G. vaginalis*), sub-dominant Fusobacteria (*L. amnionii*) and BVAB2, is restored by treatment to dominant Lactobacillaceae, where it remains until conversion. These groups rise and fall frequently, over several orders of magnitude and often together, trending upward after the penultimate menses, before recurrence was diagnosed.

**Figure 5:**
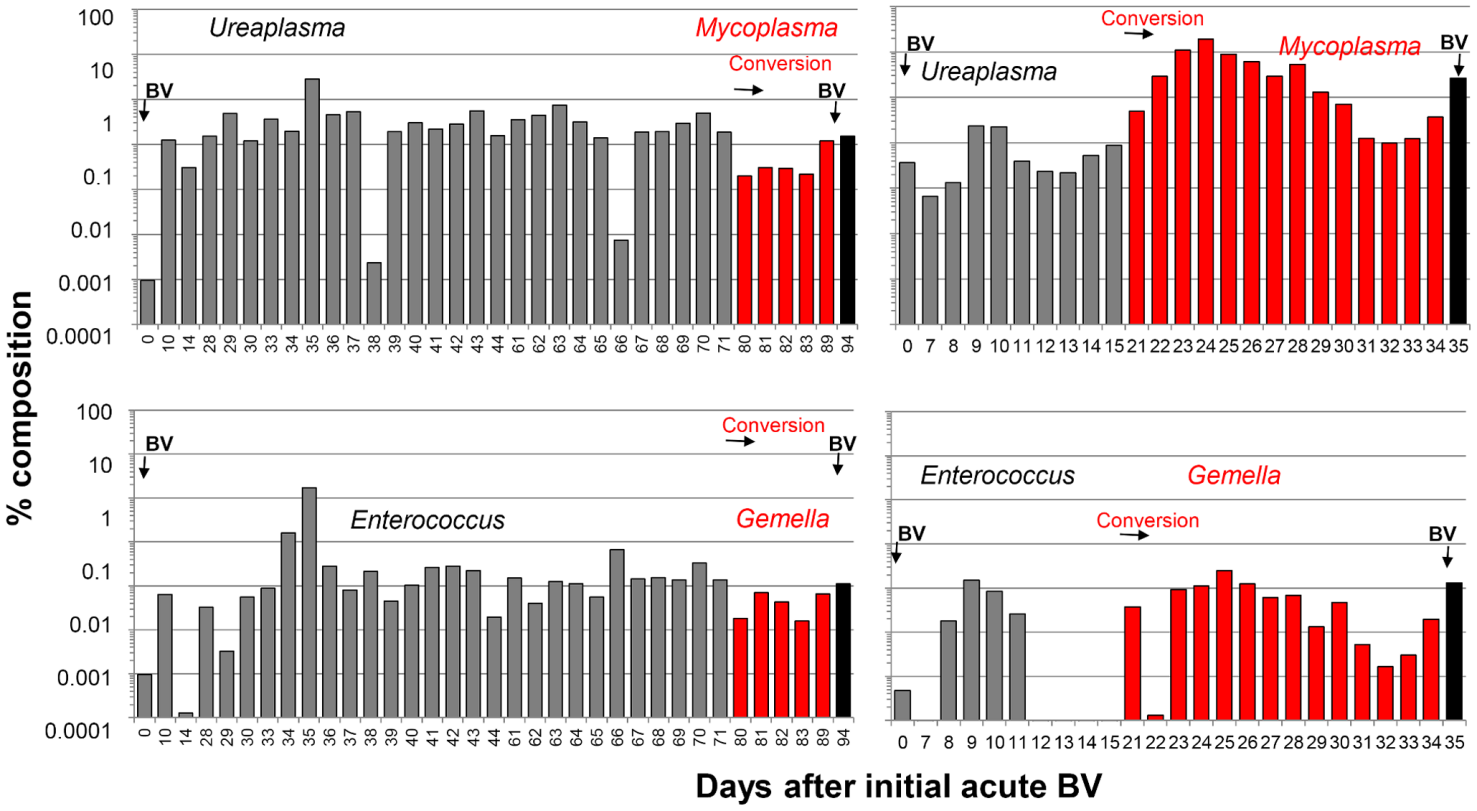
Shifts in dominant species common to conversions in P1 and P2. Panels represent qPCR data using Mycoplasmatales primers (top) or *Enterococcus* primers (bottom). Amplicons were sequenced to identify species, or in some cases were identified by their distinguishing melt curves that matched those of the sequenced products.

Conversion in P1 was coincident with species shifts in some target groups (Fig. 5). In both recurrent patients (P1 and P2), qPCR with Mycoplasmatales primers (Fig. 5, top) detected *Ureaplasma parvum* as the dominant species in pre-conversion samples, but switched to *Mycoplasma sp.* throughout conversion. Quantitative PCR with *Enterococcus* primers (Fig. 5, bottom), revealed that pre-conversion samples were dominated by *E. faecalis*, which switched to a non-target *Gemella sp*. throughout conversion. Semi-quantitative PCR (not shown) indicated that the cytolysin Cyl_lL_ gene, present in one third of clinical strains of *E. faecalis* [77], [78], was present at increasing levels preceding conversion.

**P2.** Conversion events in P2 (Fig. 3, 4) occurred immediately after her first and third menses after treatment preceding BV. The first of these was characterized by and a 100-fold increase in Actinobacteridae (*G. vaginalis*) and a somewhat later and oscillating decline in *Lactobacillus*. In P2, as with P1, there were sharp increases in Fusobacteria, (mostly *L. amnionii*), Coriobacteridae (*A. vaginae*), and BVAB2 (Fig. 4). Before conversion, P2 did not achieve the same level of reduction of multiple species compared to P1, and like P1, several groups shifted up or down together over several orders of magnitude.

**P3** and **P4** did not recur and had none of the above shifts that defined conversion in P1 and P2. In contrast to both recurring patients, P3 and P4 maintained high relative content of *Lactobacillus* throughout their post-treatment histories, and neither had dominant Actinobacteridae (*G. vaginalis*) populations at onset or during the course of study; instead, they presented with dominant *Mobiluncus* (*M. mulieris*) and *Mycoplasma* spp., respectively. *BVAB1* and *BVAB2* never rose above 0.1%, and averaged <0.003%, throughout remission. *Enterococcus* and its cytolysin Cyl_L_ gene was largely absent in P4, but were detected throughout the sampling interval, mostly at low titer, in P3 (semi-quantitative PCR, data not shown). P4 had sporadic, 1–2 day spikes of *Streptococcus* species, again, not sufficient to induce conversion. As in P1 and P2, these patients had frequent, coordinate shifts of multiple groups over orders of magnitude, but generally remaining below 1% of total titer.

## Discussion

The two most important findings from our monitoring of near-daily changes in the vaginal microbiota of five recurrent bacterial vaginosis (RBV) patients, using PB-qPCR and LB-blocked qPCR, were 1.) conversion, the loss of *Lactobacillus* and its replacement by other species, occurs well before symptomatic BV, and 2.) post-treatment samples were separable into two groups on the basis of relative *Lactobacillus* content, such that those with complete dominance (high ΔCq) were seen in patients with no or slow recurrence. These two distinct categories were also seen in a separate collection of patients. In both patient sets, post-treatment samples were Amsel negative and Nugent 0–3, suggesting that these parameters are not useful indicators for the two types of recurrence. Patients did not convert from *L. iners* to *L. crispatus* after treatment, even in those who did not recur.

Further studies with more patients are needed to pursue these trends. One potential is that ΔCq scores at post-treatment could be a new tool for the clinician to evaluate the efficacy of treatment and intervene with individualized therapy, despite the absence of symptoms. The other is that ΔCq could be used to monitor RBV patients, to allow intervention at conversion and avoid recurrence.

Conversion occurred immediately after, or possibly during menses, more than a week before symptoms, in all three episodes of acute BV, but was not observed in non-recurring patients. All conversions have the drop in *L. iners* in common. *P. timonensis* replaced *L. iners* in P1, but *G. vaginalis* was first to dominance in P2. Conversion occurred rapidly in P2, at the first post-treatment menses, perhaps foreseeable from her poor (low) ΔCq value after treatment, and perhaps suggesting that P2 hosts more virulent strains of the BV-associated anaerobes. The slower recurrence in P1 corresponded to a higher post-treatment ΔCq value, and suggests she took a different route to recurrence. This route may involve smaller, sequential perturbations after each menses, which gradually reduced dominance of *L. iners*. Samples taken during conversion in P1 and P2, or during specific days in P3 and P3 with LbRC scores <5, would likely have been described as asymptomatic BV by Nugent and Amsel criteria.

Conversion, as a potential lead-in to acute BV, raises the question of what initiates the process. Speculatively, patients that host more virulent strains, e.g. of *G. vaginalis*, may only need menses to tilt the balance toward conversion. Patients with less virulent strains may need other factors. Species showing transient increases in pre-conversion samples of P1 included *Streptococcus* sp. and *BVAB2*. Another candidate is *E. faecalis*; it is prevalent before conversion, but did not increase during conversion or acute BV. It was consistently present in P3, who did not recur in this study interval, but had a history of RBV. Non-recurrent P4 had low, intermittent levels, and it is infrequently present among patients with no history of BV. Our unpublished data indicate that vaginal *E. faecalis* strains, if beta-hemolytic, are strongly antagonistic to most vaginal *Lactobacillus* species in vitro, whereas alpha-hemolytic *E. faecalis* strains are not. That an *Enterococcus* cytolysin might play a role in BV was suggested by reports that a bacteriocin from *E. faecium* inhibited vaginal *Lactobacillus* species [79], [80]. However, this bacteriocin/cytolysin is not related in sequence, is not hemolytic [81], and we did not detect the species in our vaginal samples.

We propose a working model, that beta-hemolytic *E. faecalis*, or any species that has acquired its cytolysin operon, is responsible for initiating early changes in the vaginal microbiota that leads to conversion and BV, at least in some patients. This cytolysin is induced in response to target cells [82], in this case, erythrocytes during menses, and either directly or indirectly, contributes to the reduction of *Lactobacillus* and overgrowth of non-*Lactobacillus*. Direct reduction may involve direct lysis of susceptible species or strains of *Lactobacillus*. Indirect reduction may involve release of growth-limiting iron by hemolysis, taking away the advantage otherwise afforded by the ability of *Lactobacillus* species to thrive in an iron-poor environment [83], [84] and possibly sequester iron away from other species [85]. The cytolysin activity may precede, augment, or replace vaginolysin from *G. vaginalis*, which is hemolytic but not bacteriolytic, and varies widely in expression levels among isolates from acute BV samples [86]. The extent to which cytolysin activity is important among individuals may also depend on the degree of virulence of the non-*Lactobacillus* species (how much help do they need?), or the degree of susceptibility of specific *Lactobacillus* species. It may also depend on the severity or length of the menses, a link already established by the timing of BV with menses and its reduced prevalence among women using estrogen-based contraceptives [58], [87]–[91].

Our observation was that recurring patients were repeatedly dominated by *G. vaginalis* at acute BV episodes, whereas non-recurring patients were predominantly *Mobiluncus* (*M. mulieris*) or *Mycoplasma* sp. when acute. *G. vaginalis* sub-dominance in acute BV samples is expected from next-generation sequencing studies; for example, only 53% of 114 acute BV patients analyzed by 16S rRNA gene pyrosequencing had titers above 10% [25]. Future studies may validate that *G. vaginalis* dominance, as a subtype of BV, is associated with recurrence. Surprisingly, we are not aware of studies that address this issue. Alternatively, they may show that only specific genotypes of *G. vaginalis* correlate with rapid recurrence.

Both acute and in-remission samples were diverse, positive for many of the tested target species, and dynamic, fluctuating over many orders of magnitude in sequential samples. Many of these never rose above 1% throughout the study interval, and they tended to rise and fall in unison, often daily, as sub-dominant species. This reflects the dynamic nature of the competing species in the vaginal “ecosystem”, and is consistent with sequential variation seen in other studies using Gram staining [66]–[69], [92], species-specific qPCR [69], [70] or 16S rRNA gene pyrosequencing [24]. Despite this diversity, the intra-patient similarity among BV episodes for P1 and P2 was remarkable. Not only were there similarities in percentages (e.g., the amount of *G. vaginalis* during clinically confirmed BV episodes differed by less than 6% in P1 and 8% in P2), but frequently in species. For instance, in P1, *Megasphaera/Dialister/Veillonella* transitioned from *Megasphaera genomosp*. type1 during BV to *Veillonella* sp. during remission and back to *Megasphaera genomosp*. type1 during conversion and relapse.

The dominant *Lactobacillus* species of all five patients at acute BV was *L. iners*, which was the dominant species in post-treatment samples. The latter resemble community III of healthy women as defined by a16S rRNA gene pyrosequencing study largely by the single criteria: dominance by *L. iners* [23]. Members of this community rarely switched to other types over 4 months, notably not converting to a sustained high Nugent score, but they did have a high incidence of sporadically high Nugent scores. Community III may consist of subtypes, composed of different species present at <0.1%, still representing substantial actual titers. The subgroup composition may influence whether and how frequently these women undergo conversion. Dominance by *L. iners* is a strong risk factor for BV [93], suggesting a causal link, such as its putative lesser ability to protect the vaginal mucosa from conversion, e.g. due to its inability to produce hydrogen peroxide [94]. The opposing interpretation is that *L. iners* has been selected for among recurrent BV patients, because it is impacted less by BV treatments or because it better exploits the vaginal environment during acute BV and drives recovery [95]. Which is correct is not yet clear.

Some of the five patients were colonized with *Candida*. P2 was colonized with *C. albicans* at each clinical visit. P3 had intermittent pruritus due to culture-confirmed *Candida parapsilosis* with no recurrence of BV. P5 became culture positive for *C. albicans* at her final visit, co-incident with recurrence of BV. These observations raise the issue, but do not address, whether there are reciprocal influences between *Candida* and the vaginal bacterial microbiota. The clinical perspective, based on culture and microscopy, is that co-infections of *Candida* and BV-associated species are common (20–30% of BV patients are co-infected), but only rarely do symptoms reflecting both infections (mixed vaginitis) arise [96]. Indeed, vaginal colonization or infection with *C. albicans* did not perturb bacterial profiles, based on culture, in healthy women or in those with BV [97]. Molecular studies looking concurrently at fungal and bacterial vaginal populations are rare and preliminary. A pyrosequencing study did not show strong correlations between *Candida* colonization and dominant bacterial populations among asymptomatic women [98]. In another study, 21 patients with recurrent vulvovaginal candidiasis (RVVC) were compared to 19 healthy women using T-RFLP, to find no association of diversity or bacterial composition (notably *L. iners* versus *L. crispatus*) with RVVC [99]. Preliminary analysis (PB-qPCR) of a study in our laboratory that tracked 28 RVVC patients after they were taken off of long-term fluconazole therapy, also did not find any correlation of bacterial profiles with recurrence or acute episodes of VVC. More rigorous molecular studies, with careful clinical assessment of BV and VVC, are needed to support or refute this counter-intuitive perspective, that *C. albicans* can colonize and proliferate, indifferent to its bacterial environment.

Certainly more RBV patients will need evaluation to establish potential correlations of conversion and low post-treatment ΔCq values with recurrence. Post-treatment compositions were also identified as predictors of recurrence in a study of lesbian women [100]. But our study indicates that, pending validation, recommendations might be made at the post treatment stage (visit or self-swab), for example, that ΔCq values less than 2–3 warrant longer or more aggressive treatment, or that values in this range seen in RBV patients in remission, particularly after menses, signal conversion and a need for further treatment. Similarly, defined subgroups of acute BV profiles may be useful in predicting recurrence or deciding how aggressively to treat.

## Supporting Information

Methods S1 **Methods.**
(DOCX)

Table S1 **Characteristics of recurrent bacterial vaginosis patients.**
(XLSX)

Table S2 **Primers and programs used for PB-qPCR.**
(XLSX)

Table S3 **Percent compositions of vaginal microbiota in acute BV (Visit 1) versus post-treatment Visit 2 identified by PB-primer target groups.**
(XLSX)

Table S4 **Percent compositions of recurrent BV patients of groups targeted by PB-qPCR.**
(XLSX)
