# Supplementary material for: Longitudinal Analysis of Vaginal Microbiome Dynamics in Women with Recurrent Bacterial Vaginosis: Recognition of the Conversion Process
Source: PLoS One. 2013 Dec 20;8(12):e82599. doi: 10.1371/journal.pone.0082599 (PMC3869700; doi:10.1371/journal.pone.0082599)
Supplement: Methods S1 — Methods. (DOCX) [file pone.0082599.s001.docx]

**Supplement S1: Methods**

**Detailed patient information**

**Patient 1.** A 39-year-old married African-American woman had been seen in the WSU Vaginitis Clinic on multiple occasions for recurrent bouts of bacterial vaginosis (RBV) over a 9-year period. In the two previous years, she presented with 4 florid episodes of BV in spite of receiving maintenance suppressive prophylactic therapy with vaginal metronidazole 500-750 mg twice weekly. The last two of these four episodes was characterized by molecular methods as described below. She was enrolled as a longitudinal patient three months after discontinuing prophylactic vaginal metronidazole and after being asymptomatic in full clinical remission for seven months. At enrollment she presented with florid BV. She received daily metronidazole suppositories 750 mg and returned 10 days later in full remission. No additional antibacterial therapy was prescribed and the self-obtained vaginal 35 swabs were collected over the next three months, during which time she remained asymptomatic, until returning on day 94 with recurrence of symptomatic BV. In the interim 90 days she was seen three times in follow up in the clinic and found to be in full remission, the last visit some three weeks prior to the documented recurrence. At all three follow up visits, her measured vaginal pH was within normal limits, no clue cells were detected and whiff tests were negative. Self-obtained swabs were suspended in 2 mL sterile saline, and kept refrigerated until being returned to the clinic on a weekly basis.

**Patient 2.** A 26-year-old African American female similarly was enrolled with presentation with acute symptomatic BV. She received oral metronidazole 500 mg bid for 7 days and returned one week later asymptomatic. Thereafter she self-obtained vaginal swabs daily, but experienced Amsel and Nugent symptomatic recurrence 35 days after enrollment. She was culture-positive for *Candida albicans* at all 3 visits. This second episode of BV was treated with 500 mg metronidazole suppositories, 2 per day for 7 days, after which she recovered well off therapy, obtaining daily swabs until day 93. She had a prolonged menses, and resumed daily swabs on day 122 without symptoms until she reported symptoms at day 152, but did not return to the clinic for confirmation. Patient 2 collected her self-swab in 2 mL lysis solution (10% SDS, 10 mM Tris, pH 8, and 10 mM EDTA) and stored her swabs at room temperature until returning to the clinic for her 1 month follow up appointment.

**Patient 3.** A 35-year-old African American woman with history of recurrent BV presented with florid signs of BV and Nugent score of 10. She was treated with 2% clindamycin for 7 days and returned three weeks later in full remission and a Nugent score of 0. No further therapy was advised and daily vaginal swabs recommended. She returned 6 weeks later without symptoms and still in full remission by Nugent and Amsel criteria. She was followed and seen monthly over the next 6 months and remained in clinical remission with regard to BV but had intermittent pruritus due to culture-confirmed *Candida parapsilosis* co-infection. Patient 3 collected her self-swabs in 2 mL lysis solution. In all, 125 vaginal specimens were obtained, however the first 28 self-swabs were excluded from analysis due to problems during DNA extraction.

**Patient 4.** Patient 4 was a 24-year-old African American woman with history of recurrent bouts of BV. Married and heavy smoker, she presented recurrence of BV, confirmed on physical examination, by the presence of all 4 Amsel criteria and Nugent score of 9. She was treated with tinidazole 500 mg bid for 7 days. She returned one week later after completing therapy, and was entirely asymptomatic with normal physical findings, pH 4.2, negative amine test and normal flora morphotypes. She agreed to collect daily vaginal swab samples to monitor microbiome changes. Swabs were obtained over 30 days in absence of symptoms. Patient 4 collected swabs in 2 mL sterile saline as described for Patient 1.

**Patient 5.** A 32-year-old African-American woman presented with florid BV, fulfilling all four Amsel diagnostic criteria and a Nugent score of 10. She received 7 daily 500 mg metronidazole vaginal suppositories. She returned 21 days later asymptomatic in clinical remission; however, saline microscopy revealed mixed flora and her Nugent score was 4. She obtained 22 daily vaginal swabs and returned 56 days later for a second post-treatment visit complaining of itching and discharge. Although culture was positive for *C. albicans*, she fulfilled all four Amsel criteria and had a confirmed Nugent score of 4.

**Vaginal Self Swab Instructions**

1. Partially peel open the swab package. Remove the swab. Do not touch the soft tip or lay the swab down. If the soft tip is touched, the swab is laid down, or the swab is dropped, use a new swab.
2. Carefully insert the swab into the vagina about 2 inches (5 cm) past the introitus (vaginal opening) and gently rotate the swab for 10 to 30 seconds. Make sure the swab touches the walls of the vagina so that moisture is absorbed by the swab and then withdraw the swab without touching the skin.
3. While holding the swab in the same hand, unscrew the cap from the tube. Do not spill the contents of the tube. If the contents of the tube are spilled, use a new tube.
4. Carefully break or cut the swab to fit in the tube. Immediately discard the top portion of the swab shaft.
5. Tightly screw the cap onto the tube. Date the tube and circle whether any of the listed conditions apply for the day (B = blood, O = odor, D = discharge, S = sex)
6. Store at room temperature (lysis solution) or refrigerate (saline). Bring accumulated tubes to next clinic visit. If symptoms arise, contact the clinic for a follow-up visit.

Modified from <http://www.gen-probe.com/pdfs/pi/502259-EN-RevA.pdf>
